# Antiproliferative Effects of St. John’s Wort, Its Derivatives, and Other *Hypericum* Species in Hematologic Malignancies

**DOI:** 10.3390/ijms22010146

**Published:** 2020-12-25

**Authors:** Alessandro Allegra, Alessandro Tonacci, Elvira Ventura Spagnolo, Caterina Musolino, Sebastiano Gangemi

**Affiliations:** 1 Division of Hematology, Department of Human Pathology in Adulthood and Childhood “Gaetano Barresi”, University of Messina, 98125 Messina, Italy; cmusolino@unime.it; 2 Clinical Physiology Institute, National Research Council of Italy (IFC-CNR), 56124 Pisa, Italy; atonacci@ifc.cnr.it; 3 Section of Legal Medicine, Department of Health Promotion Sciences, Maternal and Infant Care, Internal Medicine and Medical Specialties (PROMISE), University of Palermo, Via del Vespro, 129, 90127 Palermo, Italy; elvira.ventura@unipa.it; 4 School and Operative Unit of Allergy and Clinical Immunology, Department of Clinical and Experimental Medicine, University of Messina, 98125 Messina, Italy; gangemis@unime.it

**Keywords:** *Hypericum*, St. John’s wort, hypericin, hyperforin, leukemia, lymphoma, apoptosis, multidrug resistance, photodynamic therapy

## Abstract

*Hypericum* is a widely present plant, and extracts of its leaves, flowers, and aerial elements have been employed for many years as therapeutic cures for depression, skin wounds, and respiratory and inflammatory disorders. *Hypericum* also displays an ample variety of other biological actions, such as hypotensive, analgesic, anti-infective, anti-oxidant, and spasmolytic abilities. However, recent investigations highlighted that this species could be advantageous for the cure of other pathological situations, such as trigeminal neuralgia, as well as in the treatment of cancer. This review focuses on the in vitro and in vivo antitumor effects of St. John’s Wort (*Hypericum perforatum),* its derivatives, and other *Hypericum* species in hematologic malignancies. *Hypericum* induces apoptosis in both myeloid and lymphoid cells. Other *Hypericum* targets include matrix metalloproteinase-2, vascular endothelial growth factor, and matrix metalloproteinase-9, which are mediators of cell migration and angiogenesis. *Hypericum* also downregulates the expression of proteins that are involved in the resistance of leukemia cells to chemotherapeutic agents. Finally, *Hypericum* and its derivatives appear to have photodynamic effects and are candidates for applications in tumor photodynamic therapy. Although the in vitro studies appear promising, controlled in vivo studies are necessary before we can hypothesize the introduction of *Hypericum* and its derivatives into clinical practice for the treatment of hematologic malignancies.

## 1. Introduction

**Figure 1:**
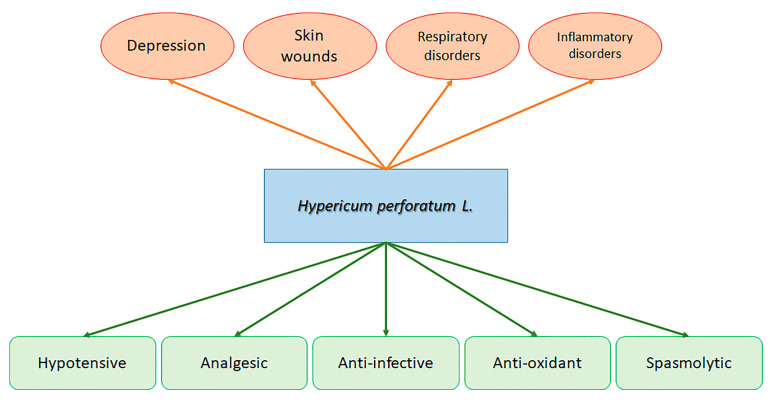
Therapeutic usefulness (**red**) and ascertained beneficial properties in (**green**) of *Hypericum perforatum* L.

The *Hypericum* genus is broadly allocated and is presently believed to include over 500 species. The Mediterranean region is a hot spot for *Hypericum* spp.; however, several species are present in America and Asia, and many are endemic species [1]. Among the multitude of *Hypericum* species, *H. perforatum* L. (Clusiaceae), generally named St. John’s wort (SJW), is one of the most relevant and notorious species. *Hypericum perforatum* L. is a perennial plant. It is widely planted in Europe, and extracts of its leaves, flowers, and aerial elements have been employed for many years as therapeutic cures for depression, skin wounds, and respiratory and inflammatory disorders (Figure 1) [2,3,4]. It also displays an ample variety of other different biological actions, such as hypotensive, analgesic, anti-infective, anti-oxidant, and spasmolytic abilities (Figure 1) [3,4,5,6,7]. However, recent investigations highlighted that this species could be advantageous for the cure of other pathological situations, such as trigeminal neuralgia [8,9], and in the treatment of cancer [10,11].

The genus *Hypericum* includes other species utilized as medicine in diverse regions of the world. Relevant therapeutic applications have been reported for *H. polyanthemum, H. drumondii, H. mysorense, H. patulum*, and numerous other components of the genus *Hypericum* as well as several of their phytocomponents [12,13,14,15]. In fact, to date, more than 900 chemical elements have been recognized from *Hypericum* species, comprising phloroglucinol products, naphthodianthrones, xanthones (principally represented by hypericin and pseudohypericin as well as protohypericin and protopseudohypericin), flavonoids (such as astilbin, rutin, miquelianin, hyperoside, quercetin, quercitrin, isoquercitrin, and I3,II8-biapigenin), a group of phloroglucinol derivatives (such as hyperforin, adhyperforin, hyperfirin, and adhyperfirin), and other phenolic elements (such as chlorogenic acid, 3-O-coumaroylquinic acid, and terpenoids) [16,17,18,19,20].

Hypericin has been recognized among the most effective elements. Both in vitro and in vivo studies demonstrated that the red-colored pigment hypericin was primarily responsible for the therapeutic actions of *Hypericum* [21,22]. Hypericin operates as an anti-depressant drug through various systems, such as 5-hydroxytryptamine1 receptor and *γ*-aminobutyric acid A receptor binding, the inhibition of glutamate release, and the reduction of dopamine-*β*-hydroxylase [23]. This substance operates by decreasing the viral infectivity and proliferation [24]. Hypericin is also a multi-layered participant in cell signaling [25]. Hypericin reduces the amounts of epidermal growth factor receptor tyrosine kinase, insulin receptors, and protein kinase C, abolishes the phorbol-12-myristate-13-acetate and tumor necrosis factor *α* (TNF-*α*)-caused stimulation of nuclear factor *κ*-light-chain-enhancer of activated B cells (NF-*κ*B), and inhibits the synthesis of prostaglandin-E2 and interleukin 6 [26,27,28].

Finally, as mentioned before, the antiproliferative capacities of *Hypericum* species and their derivatives have been assessed in several cancer cell lines, presenting data on the bioactivity of single component and combinations, such as petrol, methanol, ethanol, dichloromethane, ethyl acetate, and petrol ether extracts.

The purpose of this review is to analyze the data present in the literature on the antitumor capacity of St. John’s Wort, its derivatives, and other *Hypericum* species against cells of acute and chronic, myeloid, and lymphoid hematologic malignancies [29,30].

## 2. Antiproliferative Activities of *Hypericum* Derivatives on Myeloid and Lymphoid Cells

*Hyperforin* (HF) is a polyprenylated acylphloroglucinol derivative. The HF framework is developed from isobutyryl CoA and three molecules of malonyl-CoA by isobutyrophenone synthase [31].

Studies have shown that extracts of St.John’s wort, including HF, reduce the proliferation of human and animal tumor cells. Data from clinical and biological experimentations indicated that in vitro, HF decreased the growth of leukemia K562 cells (a human immortalized myelogenous leukemia cell line) [32], while in vivo, hyperforin reduced the proliferation of autologous MT-450 breast carcinoma in immunocompetent animals without any symptoms of acute toxicity. HF employment reduced the proliferation of these cells in animal models without any symptoms of acute toxicity [33]. Recent studies reported that HF presented antitumor actions via the stimulation of programmed cell death in tumor cells [26] and the reduction of angiogenesis [33,34,35].

In 2003, Hostanska et al. demonstrated that HF stopped the growth of human myeloid leukemia cell lines [36]. Studies have reported that HF provoked a cell cycle block in the G1 and G2 phases in acute myeloid leukemia (AML) cells differentiated by distinctive French–American–British (FAB) subtypes specifically HL-60 (M2), U937 (M5), NB4 (M3), and OCI-AML3 (M4) cells [37]. The absence of a connection between the p53 status and the reduction of cell growth persuasively proposed that HF functions via a p53-independent system in these AML cell lines.

A hyperforin-induced reduction of proliferation was associated with apoptotic cell death. In U937 cells, HF increased caspase-9, -8, and -3 and caused Poly ADP Ribose polymerase (PARP-1) cleavage. The pan-caspase inhibitor z-VAD-fmk and substances capable of blocking caspase-9 and -3 were able to reduce programmed cell death [33,37]. HF caused cell death in AML cells regardless of their FAB type. Peripheral blood mononuclear cells from normal subjects were not modified [37]. However, different studies demonstrated that HF did not stimulate the caspases directly [37] and that programmed cell death in U937 cells was due to an increase of pro-apoptotic Noxa and reduction of anti-apoptotic B-cell lymphoma 2 (Bcl-2), thus directly influencing the mitochondrial death pathway [37]. In K562 cells, HF administration caused caspase-dependent apoptosis through the mitochondrial pathway [38].

Activated AKT serine/threonine kinase 1 (AKT1) signaling prevented AML cells from programmed cell death [39]. AKT1 negatively controls the programmed cell death of leukemic cells via the process of phosphorylation of the B-cell lymphoma 2 (Bcl-2) family member Bad [40]. This causes Bad inactivation, as only non-phosphorylated Bad can elicit apoptosis. Studies have shown that HF reduced the concentration of the functioning configuration of AKT1 through dephosphorylation in leukemic cells. Researchers demonstrated that HF directly reduced the AKT1 kinase activity. As an effect of AKT reduction, HF reduced the phosphorylation of Bad and, thus, stimulated its activation [39].

**Figure 2:**
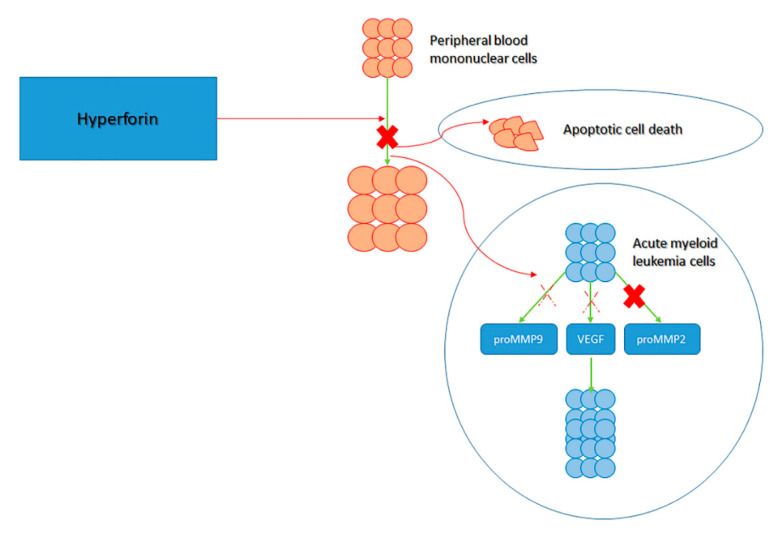
Activity of hyperforin (HF). HF is known to block the proliferation of peripheral blood mononuclear cells via: apoptotic cell death, inhibition of the promatrix metalloproteinase-2 (MMP-2) production by acute myeloid leukemia (AML) cells and inhibit proMMP-9 and vascular endothelial growth factor (VEGF) that would ordinarily promote acute myeloid leukemia (AML) cell growth.

Alongside the activation of apoptotic dynamics, other mechanisms have been identified. Acute myeloid leukemia cells generate vascular endothelial growth factor (VEGF), which, in turn, promotes AML cell proliferation [41]. The delivery of VEGF and promatrix metalloproteinase-2 (MMP-2) by primary AML cells was reported. While HF did not modify the concentrations of promatrix metalloproteinase-2 and VEGF transcripts, it did block the generation of proMMP-2 and slightly reduced the concentrations of VEGF and proMMP-9. Surprisingly, HF caused the production of a shortened configuration of MMP-9, the importance of which in AML remains to be explained (Figure 2) [41,42].

Regarding the antitumor action of HF on lymphoid cells, the actions of HF extracted from *Hypericum perforatum* were studied ex vivo on cells from B-CLL patients [43]. HF was shown to stimulate the programmed cell death of B-CLL cells. Apoptosis increased significantly after 24 h of treatment with concentrations of HFX at 0.5 mg/mL; then, it slightly decreased by 48 h. Peak apoptosis was observed with a concentration of 5 mg/mL HF (about 9 mM).

HF also caused the reduction of Bcl-2 and Mcl-1. HF also decreased two proteins that are increased in B-CLL subject cells: the cell cycle inhibitor p27 kip1 and the nitric oxide (NO) synthase of type 2. This substance was associated with a decrease in the generation of NO recognized to be antiapoptotic in B-CLL cells. Inhibiting actions of the general caspase inhibitor z-VAD-fmk demonstrated that the HF-stimulated programmed cell death of B-CLL cells was essentially caspase-dependent. Normal B lymphocytes derived from healthy subjects appeared less susceptible to HF-caused apoptosis than did B-CLL cells [43].

A study examined the effect of the increase on the pro-apoptotic effect of HF on the cells of CLL subjects and the MEC-1 cell line (a cell line derived from B-CLL through prolymphocytoid transformation). They demonstrated that the augment in Noxa expression is a time- and concentration-dependent consequence of HF arising without modification in Noxa mRNA concentrations [42]. A post-translational control was proposed as the ability of HF to block proteasome activity in CLL cells.

Noxa silencing by small interfering RNA (siRNA) decreases programmed cell death to an extent. The administration of HF, which has no consequence on the production of the prosurvival protein Mcl-1, provokes the interaction of Noxa with Mcl-1 and the disconnection of Mcl-1/Bak complex, showing that increased Noxa dislocates the proapoptotic protein Bak from Mcl-1. This action is associated with Bak stimulation, which is associated with the delivery of apoptogenic elements from the mitochondria. These results suggest that increasing Noxa is one of the systems by which HF induces CLL cell programmed death [44].

In addition to the triggering of apoptotic dynamics, other antileukemic mechanisms are possible. For instance, in CLL cells, HF is capable of reducing the generation of two controllers of angiogenesis, VEGF and metalloproteinase-9 [34].

Particularly interesting is the study of the possible antileukemic mechanisms of hypericin, which is a different derivative of *Hypericum.* The naphthodianthrone configuration of hypericin is similar to the anthraquinone ring configuration correlated with certain anticancer drugs, including mitoxantrone. Mitoxantrone operates on the DNA topoisomerase II (topo II) to stabilize the enzyme in covalent complexes with DNA in the course of its catalytic phase [45]. These cytotoxic alterations are generally denoted as “cleavable complexes”. As with other topo II “poisons”, such as amsacrine or etoposide, the stabilization of cleavage complexes can promote recombination, disturb replication fork progression, and activate tumor programmed cell death.

Initial data showed that hypericin reduced the DNA relaxation activity of topoisomerase II, which prompted investigators to examine the system of enzyme inhibition [45]. Rather than stabilizing the enzyme in cleavage complexes, hypericin blocked the topo II from proceeding to DNA cleavage. Several studies suggested that hypericin is a formidable antagonist of cleavage complex steadiness by drugs, such as amsacrine and etoposide. This antagonism appears to be due to the capacity of hypericin to alter or intercalate the DNA structure, thus preventing topo II DNA cleavage. Supporting its non-DNA injuring, the catalytic block of topo II, hypericin, was equitoxic to HL-60 and HL-60/AMSA cells, with a half-maximal inhibitory concentration (IC50) value of 5.5 to 7 M in both lines. Hypericin was unable to increase DNA damage-responsive gene promoters that are stimulated by etoposide [46].

Regarding the likely antineoplastic action of other *Hypericum* derivatives, Valletta et al. evaluated the activities of diverse extracts of *H. perforatum* flowers on the proliferation and programmed cell death of a human erythroleukemic cell line. Their findings stated that extracts, isolated with diverse solvents, were able to block K562 cell proliferation and stimulate apoptotic cell death. The IC50 value was calculated to be 19 nM [47,48].

Owing to the encouraging antigrowth actions of extracts from *Hypericum* species, dozens of phloroglucinol products were also analyzed for their cytotoxic effects against HL-60 human promyelocytic leukemia cells. However, few of them demonstrated relevant effects. Certain hyphenrones extracted from the *H. henryi* and some hookeriones separated from aerial portions of *H. patulum* reduced the growth of HL60 cells (the IC50 ranged from 3.3 to 13.8 μM) [49,50].

The pro-apoptotic effect has also been demonstrated for other derivatives. Jacarelhyperol A, a substance separated from *H. japonicum*, can dose-dependently connect to myeloid leukemia cell differentiation protein-1 (Mcl-1), Bcl-xL, and Bcl-2. Jacarelhyperol A caused a dose-dependent decrease in the cell viability of different types of leukemia cells, such as K 562, HL-60, and THP-1 by blocking the heterodimerization of Mcl-1 with BCL2-associated X (Bax), and Bcl-xL/Bcl-2 with Bak, with IC50 values from 1.52 to 6.92 μM [51].

Finally, six polycyclic polyprenylated acylphloroglucinols (PPAPs), identified as hyperisampsins H–M (1–6), were separated from the aerial portions of *Hypericum sampsonii*, together with five already recognized analogs (7–11). Compounds 1–7 were studied for their cytotoxic actions against a group of human tumor cell lines in vitro (HL60 and NB4), of which 3, 4, and 6 demonstrated relevant cytotoxic actions with IC50 values oscillating from 0.56 to 3.00 μM. Compound 3 stimulated leukemia cell programmed cell death, confirmed by the stimulation of caspase-3, degradation of PARP, increase of Bax, and reduction of Bcl-xl and Bcl-2 [52].

As far the antiproliferative activities of *Hypericum* derivatives on lymphoid cells, a phloroglucinol derivative holding a dihydrofuran ring, petiolin C (3), was extracted from the aerial portion of *Hypericum pseudopetiolatum var. kiusianum*. Petiolins A–C (1–3) displayed a cytotoxic effect against murine lymphoma L1210 cells (IC50, 2.5, >10, and 3.3 Lg/mL, respectively) [53].

In a different study, employing the Dalton’s lymphoma ascitic (DLA) cell line, the cells were observed at different time intervals during incubation in the presence or absence of the extracts. For the short-term analysis, 50% viability was reported for concentrations between 100 and 200 μg/mL for *Hypericum patulum* and 200–400 μg/mL for *Hypericum mysorense* extract. In the long-term analysis, employing the HEp-2 cells, no colony development was described exceeding a level of 1.6 μg/mL of the *Hypericum patulum* extract [54].

The in vivo antitumor action of dispensing methanol extract of *Hypericum hookerianum* stem (MEHH) against the same cell model (DLA) was established at dosages of 100–200 mg/kg body weight for 10 days [55]. The findings suggest that dispensing of the extract not only augmented the survival of mice with ascites tumors and reduced the body weight provoked by the tumor burden but also modified several hematological parameters altered in the course of tumor progression, confirming the powerful antitumor activity of the extract [55].

These results were also confirmed by a third study [56]. The data suggested that *H. japonicum* plant extracts at very small levels were strong cytotoxic factors. The dosage necessary to destroy 50% of the DLA cells for hexane extracts and petroleum ether were 38.57 and 38.89 ppm, respectively. The smallest LD50 was displayed by methanolic extract with a concentration of 60.20 ppm [56].

Finally, the cytotoxic actions of hyperatomarin isolated from *Hypericum annulatum* were evaluated in a wide group of tumor cell lines originating from lymphomas and lymphoid leukemia [57]. The group of cell lines employed in this experiment comprised cell lines from T-cell leukemia, multiple myeloma), non-Hodgkin’s lymphoma, and Hodgkin’s lymphoma. The examined substances demonstrated powerful concentration-dependent cytotoxic actions that were analogous or superior to those of the well-known antitumor drug daunorubicin. The use of hyperatomarin on human tumor cells provoked intense mono- and oligo-nucleosomal disintegration of genomic DNA with the stimulation of programmed cell death [57].

**Figure 3:**
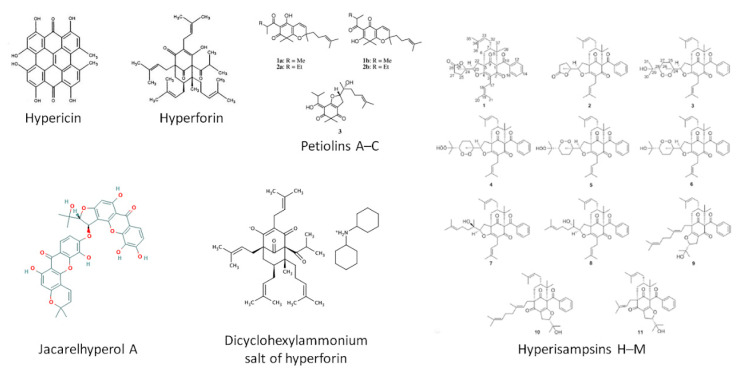
Chemical derivatives of *Hypericum*, notably hypericin, hyperforin, petiolins A–C, jacarelhyperol A dicyclohexylammonium salt of hyperforin, and hyperisampsins H–M.

The chemical structures of the main *Hypericum* derivatives are displayed in Figure 3.

### 2.1. Photochemical Features of Hypericin

Photodynamic therapy (PDT) is an antitumor treatment in which a photosensitizer, dispensed systemically or topically, is triggered by the light of an appropriate wavelength. After stimulation by light, this sensitizer employs oxygen as a substrate to generate reactive oxygen species (ROS), principally singlet oxygen. The consequent oxidative injury to the membranes and cellular structures causes a direct cytotoxic effect on tumor cells. An oxidatively balanced redox status may affect carcinogenesis by modulating DNA in particular, as well as other cellular structures. On the other hand, oxidative stress mediates the effects of many cytostatic drugs or radiotherapy by causing sublethal DNA damage and, thus, activating apoptosis. Oxidative stress is relevant in hematologic malignancies [58,59,60,61].

PDT might also provoke injury to the endothelial cells and blood cells, such as platelets and erythrocytes [62]. Remarkably, the therapeutic action of hypericin can be enhanced over 100-fold in the presence of light [63,64]. This occurs for the photochemical characteristics of hypericin [4]. The aromatic rings of hypericin give photochemical capacities to this plant pigment, which has a wide absorbance spectrum with maxima at 545 and 595 nm and a broad range of emission wavelengths topping at 594 and 640 nm [65,66,67].

The hypericin molecule harbors extended *π*-orbital electrons. After light exposure, the *π*-orbital electrons become excited to produce noxious singlet oxygen and radical species. These unstable reactive elements provoke the oxidation of cell membrane lipids and cellular proteins in a short time (milliseconds) [68]. Photo-excited hypericin can also affect the purine nucleotides in DNA [69]. These damages provoke mitochondrial injury, cytostasis, programmed cell death, and necrosis through composite systems, such as a decrease of cell proliferation signal transduction and the stimulation of cell death pathways, decrease in ATP production, and reduction of protein kinases [70,71,72,73,74,75].

Although it has been reported that the exposure of hypericin to light causes a transmission of energy to the adjacent oxygen molecules generating singlet oxygen, subsequent experiments demonstrated that hypericin was toxic even in conditions when no oxygen was accessible [76]. Studies proposed that a different light-driven chemical process named “proton transfer reaction” might be accountable for the toxic action of HF. Proton transfer reactions happen when a proton proceeds for a brief space between adjacent oxygen atoms of a molecule.

The toxic action of hypericin may be increased by a reduction of pH in its milieu [76]. Mirossay et al. evaluated the actions of three inhibitors of essential systems accountable for intracellular pH control on hypericin phototoxicity: 5-(*N*,*N*-dimethyl)-amiloride (DMA), an inhibitor of Na+/H+ exchange; N-ethylmaleimide (NEM), an inhibitor of H+-ATPase; and omeprazole (OME), an inhibitor of H+K+-ATPase [77]. Their findings demonstrated that the action of hypericin was considerably increased by DMA and NEM in a leukemic CEM cell line (acute lymphoblastic leukemia cell line). On the contrary, OME had no relevant action on hypericin cytotoxicity. Data sustain the possibility that the excited-state proton transfer and the subsequent acidification of hypericin milieu could have an essential responsibility in the biological action of hypericin [78].

The PDT efficiency of hypericin under diverse circumstances was evaluated in a P388 mouse lymphoma model [78]. Analysis of the PDT effectiveness and tissue allocation suggested that PDT effectiveness was more determined by the plasma levels than by the tumor drug concentration. Fluorescein dye exclusion demonstrated the absolute microvascular occlusion in the tumor and adjacent skin instantly after successful PDT treatments, while only a partial vascular occlusion was reported after non-efficacious PDT usage. Studies have found that tumor hypoxia, caused by hydralazine and augmenting tumor oxygenation attained by nicotinamide, could appreciably alter the efficacy of diverse PDT protocols. These findings propose that tumor vasculature alteration might be the main mechanism of hypericin-caused PDT action. The presence of this powerful different vascular action likely explains the incapacity of substances that are able to modify tumor oxygenation to change the tumor response after PDT with hypericin [78].

A study also evaluated the phototoxic and apoptosis-provoking capability of Pseudohypericin (PH) with respect to hypericin in a cell culture model with human leukemic lymphoma cells (Jurkat). The use of both photoactivated hypericin and PH provoked a dose-dependent reduction of cell growth, while non-photoactivated hypericin and PH had no action at the concentrations tested. The half-maximal inhibitory concentration (IC50) of hypericin was minor with respect to PH. In an apoptosis assay, the study demonstrated a dose-dependent augmentation of DNA fragmentation after the administration of both photoactivated hypericin and PH. The cytotoxic effects of PH should be evaluated during systemic treatment with *Hypericum* extracts, since PH is about two-times more plentiful than hypericin in *Hypericum perforatum* [79].

### 2.2. Drug Interactions. Effects of Hypericum Derivatives on Mechanism of Multidrug Resistance

Numerous findings support the possibility that several drugs have interactions with *Hypericum* and its derivatives, and important interactions have been reported with drugs, such as antidepressants, anxiolytics, antimigraine, hypoglycemic, muscle-relaxing agents, anticonvulsants, anesthetics, immunosuppressants, contraceptives, drugs used in addicted subjects (e.g., methadone), drugs acting on the respiratory system, and antimicrobic medicines and cardiovascular drugs [80]. Well-described SJW interactions comprise decreased blood cyclosporin levels, lethargy when SJW was administered with serotonin reuptake inhibitors, unwanted gravidities in women while assuming oral contraceptives and SJW, and decreased plasma drug levels of antiretroviral drugs. However, the biological interactions with other substances have yet to be thoroughly investigated, including with anticancer drugs, such as irinotecan and doxorubicin [81,82].

In an unblinded, randomized crossover study, cancer patients were treated with irinotecan (a known substrate for CYP3A4 employed in the treatment of colorectal cancer) in the presence and absence of SJW [81]. The researchers found that the plasma levels of the active metabolite SN-38 decreased by 42% following SJW cotreatment. Consequently, the degree of myelosuppression was substantially worse in the presence of SJW.

New findings proposed that drug interactions provoked by St. John’s wort derivatives could be explained through interaction with the P-glycoprotein (Pgp) or cytochrome P450 system. These data derive in part from the study of the action of *hypericum* and its derivatives on drugs used in the therapy of HIV infection. Hypericin is employed to inactivate numerous enveloped viruses present in human blood and to cure acquired immunodeficiency syndrome (AIDS) in patients [83,84].

However, in vitro experimentations propose that short-term exposure with compounds such as hypericin or extracts of SJW provoked a greater uptake or influx of ritonavir. Conversely, the long-term exposure of substances, such as hyperforin, demonstrated an increased expression of CYP3A4 mRNA, and long-term exposure of hypericin caused greater MDR-1 mRNA expression in Caco-2 cells. Therefore, such substances can operate as inhibitors or inducers. An augmentation in plasma drug concentration is possible during the concomitant administration of SJW and prescribed drugs. However, the prolonged intake of SJW supplementation followed by drug administration may cause subtherapeutic concentrations. Therefore, clinical consequences of such drug interactions depend on a variety of elements, such as the dosage, frequency, and timing of the SJW intake, dosing regimen, and route of drug administration [85,86,87].

Interaction with Pgp including stimulation, blocking, and induction can cause modified plasma concentrations of Pgp substrates [88]. Weber et al. evaluated the modulatory abilities in VLB cells (a human lymphocytic leukemia cell line expressing Pgp) and in PBCEC cells (porcine brain capillary endothelial cells). The substance, as with some of the investigated components, regulated the carrying of Pgp at micromolecular concentrations [89].

Several studies recognized the function performed by diverse *Hypericum* derivatives in the onset of resistance.

Hyperforin was demonstrated to be a powerful inducer of hepatic and intestinal cytochrome P450 monooxygenases (CYP450), mainly CYP2C9 and CYP3A4, and intestinal Pgp [90], via connecting and triggering the nuclear orphan receptor pregnane X receptor (PXR) [91]. Hyperforin appeared to be the principal reason for a relevant decrease in the plasma levels of several drugs after co-dispensation with SJW.

Hypericin stimulates the production of two ABC transporters. The first is the well-known multidrug resistance-associated protein 1 (MRP1), while the second is the breast cancer resistance protein (BCRP). As cisplatin (CDDP) and mitoxantrone (MTX) are possible substrates of ABC transporters, Jendželovská et al. evaluated the action of 24 h of hypericin treatment on the toxic effects of CDDP and MTX in HL-60 promyelocytic leukemia cells and ABCG2-over-expressing the cBCRP subclone [89].

CDDP cytotoxic action was reduced by hypericin pre-treatment in A2780cis and A2780 and MTX cytotoxic action in HL-60 cells. In contrast, hypericin increased MTX-caused death in cBCRP cells. Remarkably, hypericin did not re-establish cell growth in rescued cells. However, hypericin did augment the production of the MRP1 transporter in A2780 and A2780cis cell lines, suggesting the activity of hypericin on specific resistance mechanisms. These data suggest that hypericin may be the substratum of the BCRP transporter. Hypericin might be able to alter the efficacy of antitumor drugs [92].

Similar considerations can be made for different drugs, such as imatinib. Imatinib has been recognized as a powerful inhibitor of the Bcr-Abl tyrosine kinase. Imatinib has been reported to be very efficacious for the therapy of Philadelphia chromosome-positive chronic myelogenous leukemia (CML). A critical interference of *Hypericum* on the metabolism of imatinib was reported. Imatinib is greatly metabolized by the cytochrome P450 (CYP) enzyme system.

Frye et al. studied the actions of SJW on imatinib pharmacokinetics. SJW augmented imatinib clearance by 43%. The imatinib half-life and maximum concentration (Cmax) were also considerably reduced. *N*-desmethyl-imatinib Cmax was augmented through SJW dosing. These findings suggest that SJW enhances imatinib clearance. Thus, subjects taking imatinib should avoid taking SJW. The simultaneous use of enzyme inducers, containing SJW, may need higher levels of imatininb to preserve the clinical efficacy [92].

## 3. New By-Products and Hematologic Malignancies

Novel chemical derivatives have been discovered with enhanced solubility and stability and more powerful action, such as aristoforin [93].

Aristoforin (*O*-(carboxymethyl)-HF) is extremely constant and more solvable in water than HF. It maintains the biological functions of HF and particularly the antitumor abilities against solid cancers, but it is less toxic in animal models [94]. Aristoforin has a powerful inhibitory action on the in vitro growth of MT-450 tumor cells [94]. In the thoracic duct ring, both HF and aristoforin reduced lymphatic capillary outgrowth. Both substances were able to decrease tumor-provoked lymphangiogenesis in an animal model [93]. Clearly, preclinical studies on the sensitivity of bone marrow progenitors and normal B and T lymphocytes [95] should be performed, such as an evaluation of the efficacy of HF derivatives in CLL cells from p53 mutated patients [96].

A different hyperforin derivative is the dicyclohexylammonium salt of hyperforin (HYP-DCHA), which has been evaluated profitably. In vitro, the substance stimulates programmed cell death in animal and human tumor cell lines [28]. In vivo HYP-DCHA decreases the number of metastases in animals implanted with C-26 and B16-LU8 tumors [35,97].

The antitumor actions of HYP-DCHA were also studied in a chronic myeloid leukemia cell line [38]. HYP-DCHA showed dose- and time-dependent inhibitory actions against chronic myeloid leukemia cells, with IC50 values of 8.6 and 3.2 μM for 48 and 72 h of treatment, respectively, which were more efficient than those of the HF. HYP-DCHA administration caused a stimulation of programmed cell death as demonstrated by nuclear condensation, DNA fragmentation, and an increase of early apoptotic cells.

HYP-DCHA determined the dissipation of the mitochondrial transmembrane potential that started with the liberation of cytochrome c through the reduction of the concentration of anti-apoptotic proteins and an increase in the production of pro-apoptotic proteins. As for HF, YP-DCA administration also caused stimulation of the caspase system and PARP cleavage. The use of caspase inhibitors resulted in a reduction in programmed death induced by HYP-DCHA. After administration, cells were blocked at the G1 phase and the generation of two essential controllers correlated to cell cycle and cell death, p27Kip1 and p53, was increased [38].

The main degradation products of hyperforin in acidic aqueous solutions comprise furo-HF and furo-HF hydroperoxide [98,99,100]. Furo-HF is a powerful inhibitor of CYP3A4 but appears to be a much less potent anti-angiogenic substance than HF is [101].

Finally, a different durable derivative, acetylate HF (ace-HF), can control α-secretase-mediated amyloid precursor protein (APP) in epithelial cells transfected with APP cDNA [102]. Ace-HF displays antiproliferative actions in several tumoral cell lines, comprising the K562 cell line that is characteristic of CML. Ace-HYP’s effectiveness has been found in mouse models that are susceptible to anxiolytic and antidepressant drugs [103].

Whether these HF analogs maintain the antileukemic abilities of the parental compound in different experimental models should be determined. The action of the extract is not intense and has not been studied with respect to standard drugs. Separation of the elements of this extract may be used to detect encouraging active constituents.

## 4. Conclusions and Future Perspectives

Several anti-tumor drugs have relevant side effects, and some tumors are drug resistant. Consequently, new anti-tumor substances are needed in pharmaceutical development. Natural products are the main sources of bioactive drugs and will continue to supply the most candidates for novel drugs for hematological malignancies [93,104,105,106,107].

The studies mentioned above justify the interest in the use of *hypericum* and its derivatives in hematologic malignancies. However, numerous elements need to be kept in mind, such as interference with other antineoplastic drugs, as reported above. When pondering the importance of hyperforin for clinical purpose, numerous aspects could limit its applicability, such as interference with the clearance of xenobiotics or sensitivity of the phloroglucinol to oxygen, light, and aqueous solvents [93].

However, other aspects of the effects of *Hypericum* and its derivatives will have to be investigated, and, in the future, a particularly promising research direction relies on the relationship between *Hypericum* and the control of gene expression. Several results report that microRNAs (miRNAs) are able to modify the gene expression either by the aging of messenger RNA or translational repression. Multiple studies have demonstrated that miRNAs participate in the regulation of hematologic malignancies [108,109,110].

Studies demonstrated that plant miRNAs are existent in the sera and tissues of different animals and that these exogenous plant miRNAs are essentially assumed via food intake [111]. Galla et al. characterized mature miRNAs along with their precursors and potential targets in *Hypericum.* A computational in silico prediction of structure, in combination with an in vitro validation, allowed them to identify seven pre-miRNAs, including miR-156, miR-166, miR-390, miR-394, miR-396, and miR-414. They demonstrated that *H. perforatum* flowers share highly conserved miRNAs and that these miRNAs potentially target dozens of genes with a wide range of molecular functions [112]. In a different study, the authors investigated cross-kingdom gene regulation via miRNAs of *H. perforatum* flower dietetically absorbed in an in silico approach to define potential biomarkers for cancer. They demonstrated that some of these miRNAs potentially have a significant and critical tumor-suppressive role for prostate cancer [113].

In accordance with these findings, some genes controlled by miRNAs of *H. perforatum* may have a possible cancer suppressor action for tumor onset and progression. In conclusion, in recent years, phytochemical experimentations on *Hypericum* have become more frequent, with most studies focusing on phloroglucinol derivatives, owing to their structural diversity and exclusive biological abilities.

Although several substances were extracted and tested for bioactive capacity, few have received follow-up and therefore, questions still remain. For instance, few reports describe how metabolites change among plant tissues in the course of plant development and through seasonal modifications for *Hypericum* species. Metabolomic analyses that evaluate secondary metabolites of *Hypericum* species using ultra performance liquid chromatography-tandem mass spectrometry and multivariate statistics may be advantageous to establish bioactive markers. Whether *Hypericum* derivatives and HF analogs maintain the antileukemic abilities of the parental compound in different experimental models should be determined. The action of the extract is not intense and has not been studied with respect to standard drugs. Separation of the elements of this extract may help to detect encouraging active constituents.

An aspect still to be explored is the possible synergistic action exerted by different components of the SJW. The bioavailability of particular active components, such as hypericin, can be increased by associated substances (so called co-effectors) present in the SJW. The pharmacokinetic mechanisms can be physicochemical or biochemical via augmented transport to the plasma site. These sorts of interactions between substances are combined under the term “pharmacokinetic synergy”. These observations have relevant consequences for clinical use both for the efficacy and for the onset of side effects of drugs taken at the same time [114].

The vast majority of the data reported here suggest that *hypericum* and its derivatives might be very powerful agents for the treatment of hematologic malignancies. There is the possibility they possess much broader biological activities. Further studies are needed to investigate the antineoplastic capacity of these compounds.
